# Factors controlling throughfall in a *Pinus tabulaeformis* forest in North China

**DOI:** 10.1038/s41598-017-14464-z

**Published:** 2017-10-25

**Authors:** Xi Wei, Huaxing Bi, Wenjun Liang

**Affiliations:** 1 School of Soil and Water Conservation, Beijing Forestry University, Beijing, 100083 China; 2 Ji county station, Chinese National Ecosystem Research Network (CNERN), 100083 Beijing, China; 3 Beijing Collaborative Innovation Center for Eco-environmental Improvement with Forestry and Fruit Trees, 102206 Beijing, China; 4 Key Laboratory of State Forestry Administration on Soil and Water Conservation (Beijing Forestry University), 100083 Beijing, China; 5 College of Forestry, Shanxi Agricultural University, Shanxi, 030801 China

## Abstract

The factors that control throughfall in *Pinus tabulaeformis* plantations were investigated using linear and curve analyses based on direct measurements of rainfall, throughfall and stemflow from 36 rainfall events. The results showed the following: (1) there was significant spatial heterogeneity in throughfall rates in *P. tabulaeformis* plots; (2) the throughfall rate increased with increasing rainfall; and (3) the rate of increase gradually decreased. When rainfall reached approximately 25 mm, the throughfall rate stabilized. The coefficient of variation of the throughfall rate decreased with increasing rainfall, with a peak at approximately 10 mm of rainfall. The coefficient of variation of throughfall stabilized at 20%, and the coefficient of variation of the throughfall rate stabilized at 17%. A linear regression equation (R^2^ = 0.76) was derived by fitting the *P. tabulaeformis* average diameter at breast height (DBH), average tree height, average branch height, stand density, canopy thickness, canopy density, and the rainfall and throughfall rate. A highly positive correlation was found between the throughfall rate, canopy density, rainfall class and tree height (P < 0.01). By establishing a quadratic response surface model of the stand structure indicators and the throughfall rate, R^2^ was increased to 0.85 (P < 0.01). The quadratic regression analysis demonstrated a highly positive correlation between throughfall rate, canopy density and rainfall class.

## Introduction

Forests and water provide the basic materials for human survival and development, and atmospheric precipitation plays an extremely important role in forest ecosystems and the global water balance^1–4^. In forest ecosystems, rainfall is partitioned by forest canopies into throughfall (TF), stemflow (SF), and interception (I) loss, which have significant effects on the water balance and nutrient cycling in forest ecosystems^5–8^. Rainfall reaches the forest floor via TF and SF. International researchers have conducted a number of studies on these processes^9–13^. Primary forest types include coniferous forests, Chinese firs, *Q. monimotricha* forests, temperate deciduous forests, larch forests, tropical rain forests and subtropical evergreen forests^14–18^. Most studies have shown that when rainfall reaches the canopy, the canopy interception rate is 10–40%, the throughfall rate (TF/rainfall) is 60–90%, and the stemflow rate (SF/rainfall) is 0.5–14%^19–21^. TF is controlled by many factors, such as rainfall features, canopy structure characteristics, and meteorological conditions, as well as interactions among these factors^22–27^. Environmental factors are often used to estimate TF. For example, the rainfall interception ratio can be formulated as an exponential decay function of rainfall intensity, temperature and the wet canopy evaporation rate^28–30^. In fact, canopy structure characteristics such as vegetation type, tree density, average height, canopy thinning, branch height, branching patterns, canopy density and leaf area index (LAI) can affect the canopy water storage capacity, which is also important for estimating TF^31,32^.

While many studies have been conducted on canopy interception because of its influential factors, there is no method to rapidly estimate forest TF. In this paper we established relationships between TF and the related factors using two mathematical models and tried to find out the most significant contributor to TF of *P. tabulaeformis* plantations. The results can provide further information about the forest water cycle in the study areas. We have three steps to achieve the purpose.

(1)  Monitor rainfall, TF, stemflow and interception loss in study area. We expect in-depth understanding of TF of *P. tabulaeformis* plantations and the relationship between the four in a variety of circumstances.

(2)  Identify, classify and quantify the factors relevant to TF. In order to distinguish the single factor heterogeneity, we refine each factor and especially discriminate the heterogeneity of throughfall in detail under different rainfall class.

(3)  Compare all relevant factors, filter and establish model with mathematical method. We expect the most accurate model to determine the factors that have the most important impact on TF, by a variety of mathematical methods to calculate and verify.

## Materials and Methods

### Site description

The study site is located at the Mulan Forestry Management Bureau in Hebei Province (41°49′35.8″N, 117°35′31″E; elevation 1270 m), which lies in the cross connecting area of the Yinshan Mountains and Swallow Mountain(s) in the upper reaches of the Luanhe River, China. The study site is a typical rocky mountainous area of northern China. Meteorological records indicate that the long-term mean annual air temperature is 3.2 ± 0.3 °C, and February and August are the coldest and warmest months, respectively. During the study period, the average temperature was obtained from meteorological records. The main tree species were *Larix principis-rupprechtii, P. tabulaeformis*, *Betula platyphylla* and *Populus davidiana*; the understory vegetation consisted of *Rhododendron micranthum*, *Spiraea fritschiana*, *Carex lancifolia*, *Phlomis umbrosa* and *Thalictrum minus*.

### Instrumental setup and data collection

Six standard *P. tabulaeformis* sample plots were established in the study area, and the hydrological characteristics of these permanent sample plots were investigated from 2010 to 2011. The *P. tabulaeformis* stand density in the six plots constituted a gradient (2438 plants/hm^2^, 1815 plants/hm^2^, 1500 plants/hm^2^, 1080 plants/hm^2^, 928 plants/hm^2^ and 650 plants/hm^2^). The investigation included tree, shrub and grass surveys. Table 1 shows the plot characteristics, including the plot area, mean diameter at 1.3 m above the ground (DBH), mean tree height, canopy density, stand density, and mean branch height (Table 1).

**Table 1:** Stand characteristics of each sample plot in the study area.

No.	Age/y	Area/m × m	Density/trees·hm^−2^	Canopy density	Mean height/m	Mean DBH /cm	Mean branch height/m
1	33	30 × 20	2483	0.85	9.50	13.60	6.0
2	38	40 × 50	1500	0.86	12.40	15.40	5.9
3	43	20 × 20	650	0.61	13.02	17.63	5.8
4	41	40 × 50	1815	0.81	11.40	15.10	4.9
5	42	50 × 50	1080	0.73	13.26	17.79	6.1
6	43	50 × 50	928	0.79	11.19	17.23	4.1

A HOBO U30 weather station was established in the wilderness area outside the plots. Air temperature, air relative humidity, wind velocity, solar radiation and rainfall were measured and recorded every 10 minutes. In this experiment, throughfall was collected in water reservoirs constructed of iron sheets with a collection area of 0.156 m^2^ (0.2 × 0.78 m), which was equivalent to five standard rain gauges. An opening was made at the bottom and connected to 10-L plastic barrels using a plastic tube.

The canopy density was measured using a digital plant canopy imager CI-110 (USA). Fish-eye photographs were captured above the centre of each water reservoir at 4–9 points, which were evenly distributed in each plot. The photographs were taken 0.8 m above the throughfall collectors on cloudy days to avoid the effects of direct sunlight. Sky image pixels (a) and total image pixels (A) were used to remove the trunk from the image, and Photoshop CS software was used to process the binary image as follows: Canopy density (f) was calculated following (1), and the mean value for each measurement point was calculated. In addition, two water reservoirs placed outside the forest on open ground, at a distance of approximately 50–100 m, served as controls.

1 $$f=1-\frac{a}{A}$$

**Figure 1:**
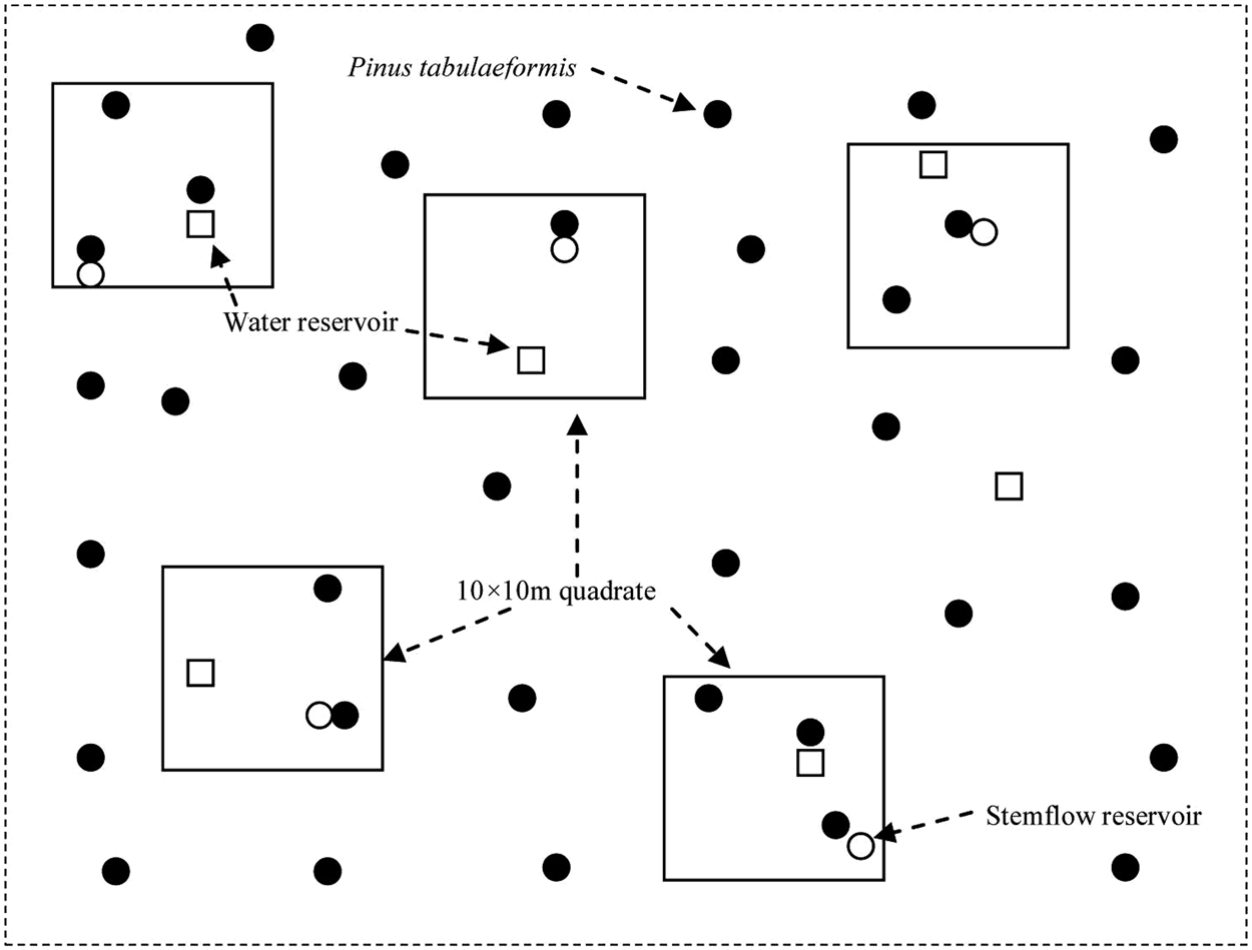
Distribution of water reservoirs in a plot.

The water reservoir devices were located based mainly on the canopy density in the sample plots (Equation 1). To ensure the measurement data were representative of the entire plot, we chose areas with dense canopy, sparse canopy and forest gaps to establish the water reservoirs because some plots contained relatively higher canopy densities. However, for stands with a relatively lower canopy density, we chose standard trees with complete canopies, as well as gap centres, to position the water reservoirs. In this experiment, there were three water reservoirs in sample plot No 1, five in No 2, three in No 3, three in No 4, three in No 5, and three in No 6 (Fig. 1).

At the same time, 10 × 10 m quadrats were established around each water reservoir in the plots. The height of the water reservoirs was at least 30 cm with an approximately 1° angle relative to the earth’s surface. The average tree height, average DBH, average branch height, average canopy thickness, canopy density and stand density were carefully investigated within each 10 × 10 m quadrat.

When considering rainfall rate and stand structure (average tree height, average DBH, average branch height, average canopy thickness, canopy density and stand density), there is a need to determine which factor is the most important. To this end, stepwise regression analysis and a quadratic polynomial equation were adopted. Then, the accuracy of each equation was compared, and a decision was reached regarding which factor was most suitable.

### Reanalysis of datasets

The SPSS stepwise regression analysis method was used, and we established the optimal linear regression equation between throughfall and stand structure variables (average tree height, average DBH, average branch height, average canopy thickness, canopy density and stand density) to determine the fit of the parameters and throughfall. To improve simulation accuracy, quadratic polynomial equations were used to determine a quadratic response surface model for throughfall and stand structure variables.

Regarding the difference in the analysis of rainfall levels, SAS software was used to perform significant difference testing and sorting of the rainfall levels associated with the throughfall rates. First, the Shapiro-Wilk normality test and Levene’s homogeneity of variance test were carried out. When the results complied with the normality and homogeneity of variance of the data, we then carried out analysis of variance and Duncan’s multiple comparisons. If any term did not match, the Kruskal-Wallis nonparametric test was applied using NPAR WAY. Based on the results of these tests, an assessment was made to identify significant differences in the rainfall level associated with the throughfall rate. If there were no significant differences in the majority of quadrats, it was assumed that the throughfall rate showed little change, and the rainfall level could be marked at the same level in the regression analysis. Based on general rainfall level division standards and data testing and rearrangement, a new level of rainfall was obtained. We then established new rainfall levels ranging from light to heavy to perform the regression analysis.

## Results

### Rainfall characteristics during the experimental period

**Figure 2:**
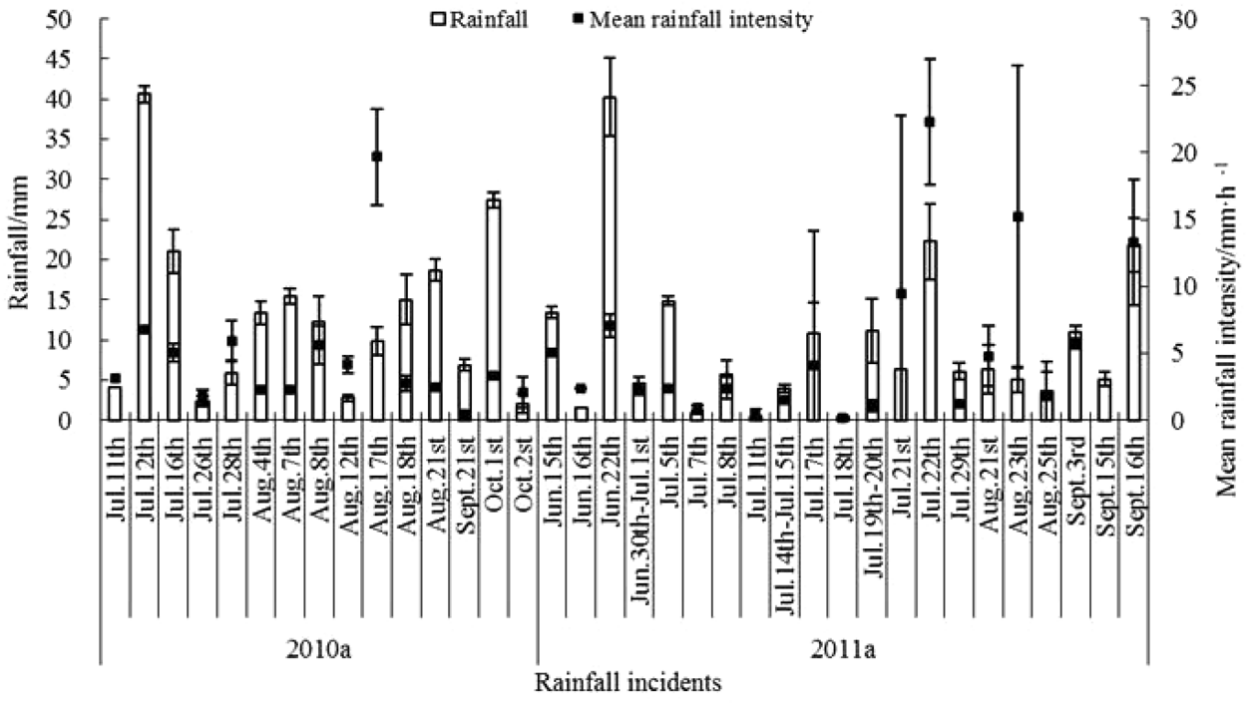
Rainfall and rainfall intensity in the study area.

To reduce error, we also observed rainfall at six locations. Rainfall at these locations was measured in an open area approximately 10 m from the plots. During the experimental period, effective rainfall was detected 36 times from July 2010 to September 2011. During the study period, there were six times when rainfall exceeded 20 mm and five times when rainfall intensity exceeded 10 mm · d^−1^ (Fig. 2).

### Throughfall and rainfall

**Figure 3:**
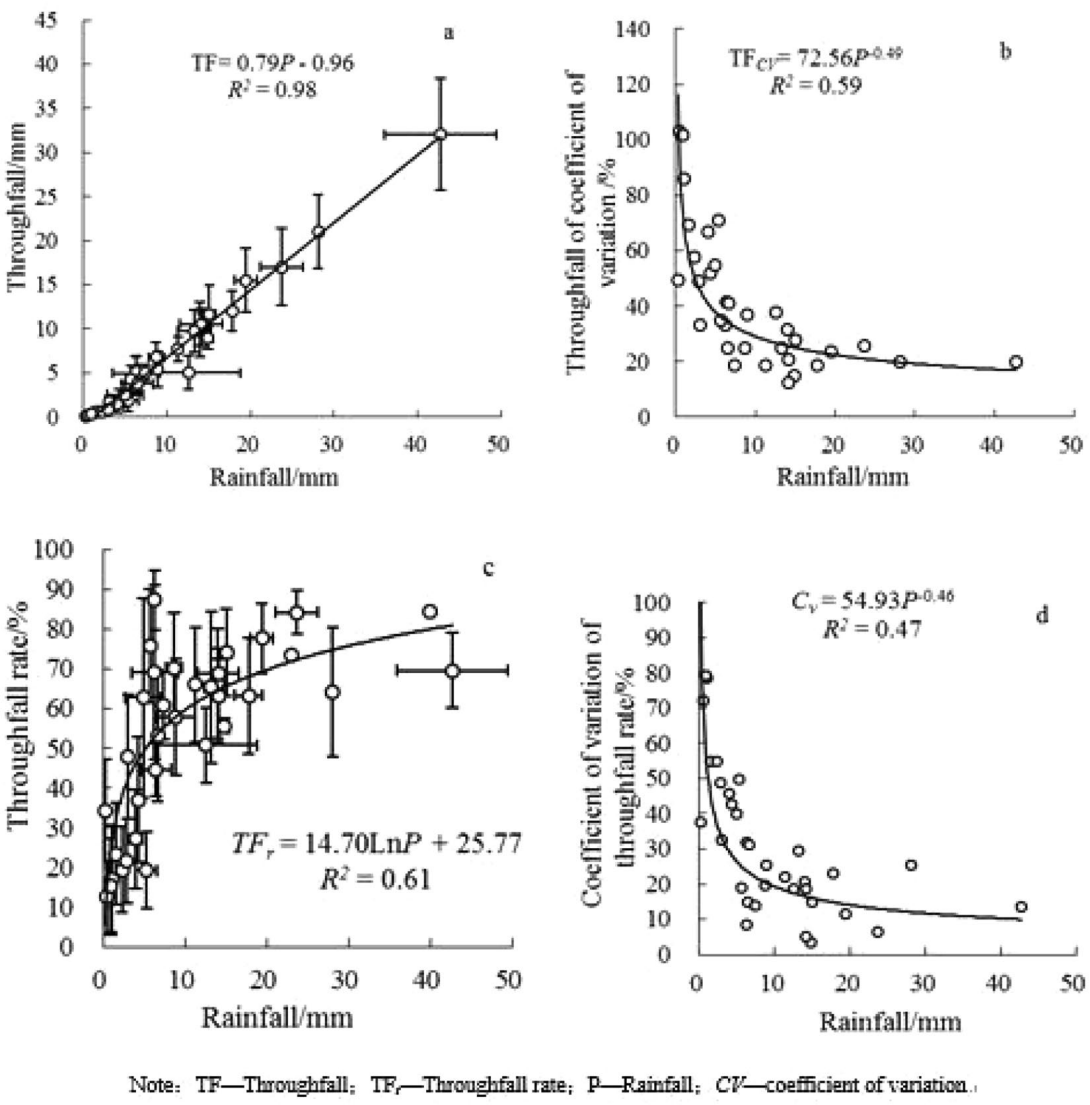
Relationship between rainfall, throughfall and throughfall rate.

Figure 3 shows the relationship between throughfall and rainfall for *P. tabulaeformis* in the study area. The figure illustrates that rainfall and throughfall have a highly positive correlation (R^2^ = 0.98) (Fig. 3a). The throughfall rate increased with rainfall, but the rate of increase gradually decreased and stabilized at approximately 75% (Fig. 3c). The interception rate stabilized at approximately 25% in this region. In addition, the throughfall rate and its coefficient of variation decreased as rainfall increased. The speed was initially fast and then became slow, with a turning point at approximately 10 mm (rainfall). The coefficient of variation of throughfall steadied at approximately 20% (Fig. 3b), and the coefficient of variation of the throughfall rate steadied at approximately 17% (Fig. 3d).

### Rainfall level evaluation

In this research, the variability of observational data at the 5-mm level of rainfall penetration was tested during the study period. Rainfall was lower than 50 mm·d^−1^ in the observed area. According to the rainfall level division standards of the China Meteorological Administration, daily rainfall in the 0~10 mm range represents light rain, 10~25 mm represents moderate rain, and 25~50 mm represents heavy rain. To determine whether there were differences in the throughfall rates of light rain, the light rain stage division was set to 5 mm, followed by a difference analysis. The same procedure was performed for moderate rain. With respect to throughfall associated with heavy rain, the rate mostly stabilized and was no longer divided (Table 2).

**Table 2:** Throughfall rate in quadrats for each rainfall class measured using rainwater collection devices for throughfall collection.

Collection device no	Rainfall class/mm·d^−1^					
	0~5	5~10	10~15	15~20	20~25	25~50
1-1	26.92 ± 12.53 d	44.99 ± 4.01 c	59.73 ± 6.68 b	62.80 ± 11.43 ab	73.46	77.49 ± 9.82 a
1-2	25.56 ± 10.5 b	46.12 ± 4.00 a	58.43 ± 7.25 c	55.36 ± 10.78 ab	79.64	75.21 ± 8.74 a
1-3	24.62 ± 5.4 b	44.34 ± 5.00 a	60.78 ± 8.54 b	50.88 ± 11.23 ab	80.09	76.76 ± 8.65 a
2-1	32.94 ± 24.88 b	58.07 ± 17.14 ab	63.00 ± 2.93 ab	63.68	76.15 ± 6.02 a	58.84
2-2	31.25 ± 25.68 c	57.85 ± 21.05 bc	70.44 ± 8.44 ab	74.01 ± 7.00 ab	83.93 ± 4.97 a	82.67
2-3	35.35 ± 26.97 b	60.82 ± 22.90 ab	66.33 ± 21.07 ab	87.51	87.13 ± 2.90 a	67.88
2-4	19.09 ± 15.75 b	53.13 ± 27.09 ab	56.17 ± 7.52 a	63.27	80.08 ± 9.57 a	60.32
2-5	36.52 ± 24.31 b	60.82 ± 18.95 ab	65.53 ± 16.90 ab	75.65 ± 4.59 ab	75.87 ± 2.28 a	68.13 ± 6.85 a
3-1	35.00 ± 22.58 a	46.36 ± 24.35 a	42.77	74.57	—	—
3-2	23.79 ± 16.30 a	35.80 ± 27.40 a	68.38	55.75	—	—
3-3	22.79 ± 15.45 a	34.70 ± 26.44 a	66.56	54.34	—	—
4-1	16.43 ± 16.21 b	55.90 ± 36.85 ab	64.41 ± 14.83 ab	73.34	84.79 ± 8.69 a	69.16
4-2	16.23 ± 14.48 a	52.40 ± 37.36 ab	66.86 ± 14.73 ab	70.25	85.25 ± 8.39 a	60.59
4-3	16.78 ± 15.50 a	50.30 ± 36.76 ab	65.43 ± 14.67 ab	71.89	84.65 ± 8.45 a	65.12
5-1	33.15 ± 14.28 b	62.83 ± 26.95 ab	74.83 ± 8.80 a	86.28	92.31	84.21
5-2	32.25 ± 10.26 b	56.24 ± 25.94 ab	70.33 ± 7.36 a	80.29	95.69	88.57
5-3	32.45 ± 10.31 b	57.34 ± 24.84 ab	71.36 ± 7.45 a	81.30	94.67	86.70
6-1	28.83 ± 17.41 a	66.30 ± 31.40 ab	86.57 ± 0.09 b	86.69	65.46	82.19
6-2	30.38 ± 15.46 a	50.87 ± 30.56 ab	78.58 ± 0.12 b	80.47	60.58	79.64
6-3	31.45 ± 15.98 a	51.76 ± 31.57 ab	80.32 ± 0.10 b	82.50	63.48	80.34

Note: Mean ± standard deviation. The same letter within a line indicates no significant difference (P < 0.05).

The analysis results showed the following (Table 2): throughfall rates in 17 of 20 small quadrats of *P. tabulaeformis* showed no significant differences when the rainfall accumulation was 0~10 mm. Therefore, we believe that throughfall rate in the *P. tabulaeformis* forest at the 0~10 mm·d^−1^ rainfall level was not significantly altered. Thus, this rainfall level (0~10 mm·d^−1^) was assigned a value of 1 to unify logistics data for regression analysis. We also found that 12 of 20 small quadrats exhibited no significant differences when the rainfall accumulation was 10~25 mm · d^−1^, representing the vast majority of events. Thus, rainfall accumulation in the 10–25 mm · d^−1^ class was assigned a value of 2. Similarly, rainfall accumulation in the 25–50 mm · d^−1^ class was assigned a value of 3 (Table 3).

**Table 3:** Throughfall rate for each rainfall class in each quadrat and corresponding stand structures in the study area.

Quadrat	Throughfall rate (%)	Average DBH *x*_1_/cm	Average height *X*_2_/m	Branch height *X*_3_/m	Canopy thickness *X*_4_/m	Stand density of plants *X*_5_/hm^2^	Canopy density *X*_6_	Rainfall class *X*_7_
1-1	32.54	13.3	11.9	5.7	6.2	3300	0.88	1
1-2	33.69	15.67	12.5	6.4	6.8	3200	0.85	1
1-3	32.12	14.45	12.3	6.1	6.5	3200	0.85	1
2-1	45.51	16.0	12.4	7.9	4.5	2000	0.93	1
2-2	43.22	15.7	12.9	8.3	4.6	1900	0.74	1
2-3	49.66	15.6	13.7	8.3	5.4	1300	0.91	1
2-4	37.02	15.9	12.6	7.6	5.0	1500	0.92	1
2-5	48.09	16.9	13.4	7.7	5.6	1400	0.78	1
3-1	41.85	15.9	13.5	8.0	5.5	5787	1.00	1
3-2	29.38	19.4	13.5	7.0	6.5	1415	1.00	1
3-3	100.00	18.0	13.1	7.2	5.9	500	0.57	1
3-4	100.00	17.9	13.1	7.5	5.6	400	0.61	1
4-1	35.42	16.7	12.4	5.7	6.8	1500	0.88	1
4-2	34.79	20.0	11.0	5.7	5.3	1273	1.00	1
5-1	45.76	16.5	12.5	6.3	6.2	1500	0.73	1
5-2	41.13	19.4	13.0	5.0	8.0	1455	1.00	1
6-1	46.10	21.3	11.9	4.8	7.1	1100	0.79	1
6-2	45.67	24.6	13.2	5.4	8.4	1050	0.84	1
1-1	61.94	13.3	11.9	5.7	6.2	3300	0.88	2
1-2	62.58	15.67	12.5	6.4	6.8	3200	0.85	2
2-1	68.40	16.0	12.4	7.9	4.5	2000	0.93	2
2-2	74.21	15.7	12.9	8.3	4.6	1900	0.74	2
2-3	78.89	15.6	13.7	8.3	5.4	1300	0.91	2
2-4	67.15	15.9	12.6	7.6	5.0	1500	0.92	2
2-5	70.63	16.9	13.4	7.7	5.6	1400	0.78	2
3-1	58.67	15.9	13.5	8.0	5.5	5787	1.00	2
3-2	62.07	19.4	13.5	7.0	6.5	1415	1.00	2
3-3	100.00	18.0	13.1	7.2	5.9	500	0.57	2
3-4	100.00	17.9	13.1	7.5	5.6	400	0.61	2
4-1	74.35	16.7	12.4	5.7	6.8	1500	0.88	2
4-2	43.70	20.0	11.0	5.7	5.3	1273	1.00	2
5-1	82.06	16.5	12.5	6.3	6.2	1500	0.73	2
5-2	55.59	19.4	13.0	5.0	8.0	1455	1.00	2
6-1	81.32	21.3	11.9	4.8	7.1	1100	0.79	2
6-2	82.35	24.6	13.2	5.4	8.4	1050	0.84	2
1-1	77.49	13.3	11.9	5.7	6.2	3300	0.88	3
1-2	78.36	15.67	12.5	6.4	6.8	3200	0.85	3
2-1	58.84	16.0	12.4	7.9	4.5	2000	0.93	3
2-2	82.67	15.7	12.9	8.3	4.6	1900	0.74	3
2-3	67.88	15.6	13.7	8.3	5.4	1300	0.91	3
2-4	60.32	15.9	12.6	7.6	5.0	1500	0.92	3
2-5	68.13	16.9	13.4	7.7	5.6	1400	0.78	3
4-1	69.16	16.7	12.4	5.7	6.8	1500	0.88	3
4-2	47.78	20.0	11.0	5.7	5.3	1273	1.00	3
5-1	84.21	16.5	12.5	6.3	6.2	1500	0.73	3
5-2	53.59	19.4	13.0	5.0	8.0	1455	1.00	3
6-1	82.19	21.3	11.9	4.8	7.1	1100	0.79	3
6-2	83.64	24.6	13.2	5.4	8.4	1050	0.84	3

Note: Number of 10 × 10 m quadrats in the plots.

### Relationship between throughfall and related factors

The throughfall rate of artificial *P. tabulaeformis* plantations was designated the dependent variable. Stand structure factors and rainfall factors closely related to the throughfall rate were chosen as independent variables (Table 3).

The throughfall associated with each rainfall level and the corresponding stand structure indicator data were analysed using stepwise regression (Table 4), and a linear regression model for the throughfall rate was fitted. The throughfall rate model was significant (P < 0.0001), and the F value was high (Table 4). The main factors affecting the throughfall rate were rainfall level (X_7_), canopy density (X_6_) and average tree height (X_2_). When the stepwise regression model included only the rainfall level (X_7_), the multiple correlation coefficient R^2^ was 0.55, and when canopy density (X_6_) was introduced, R^2^ reached 0.74. The results indicate that the rainfall level (X_7_) and canopy density (X_6_) contributed significantly to the multiple correlation coefficient; they were the main factors that affected the throughfall rate. Finally, when average tree height (X_2_) was introduced, R^2^ only increased to 0.76, indicating that the average tree height was not a significant factor affecting the throughfall rate (P = 0.11); it was the second most important factor. Thus, the optimal linear regression model for the throughfall rate (TF_r_) of the *P. tabulaeformis* forest is as follows (Equation 2):

2 $${{\rm{TF}}}_{{\rm{r}}}=3.83\cdot {{\rm{X}}}_{2}-106.07\cdot {{\rm{X}}}_{6}+11.57\cdot {{\rm{X}}}_{7}+82.33$$

**Table 4:** Stepwise linear regression analysis for the throughfall rate of the *P. tabulaeformis* forest in the study area.

	DF	Sum of Squares	Mean Square	F Value	Pr > F
Model	3	14163	4721	41	<0.0001
Error	39	4488	115		
Corrected total	42	18651			
	**Variable Estimate**	**Standard Error**	**Type II SS**	**F Value**	**Pr > F**
Intercept	82.33	34.25	665.00	5.78	0.02
Height-*X*_2_	3.83	2.36	303.29	2.64	0.11
Canopy density-*X*_6_	−106.07	12.37	8459.25	73.51	<0.0001
Rainfall class *-X*_7_	11.57	2.04	3697.28	32.13	<0.0001
**Step**	**Variable Entered**	**Variable Removed**	**Partial R-Square**	**Model R-Square (R** ^**2**^ **)**	
1	*X*_6_		0.55	0.55	
2	*X*_7_		0.19	0.74	
3	*X*_2_		0.016	0.76	

**Figure 4:**
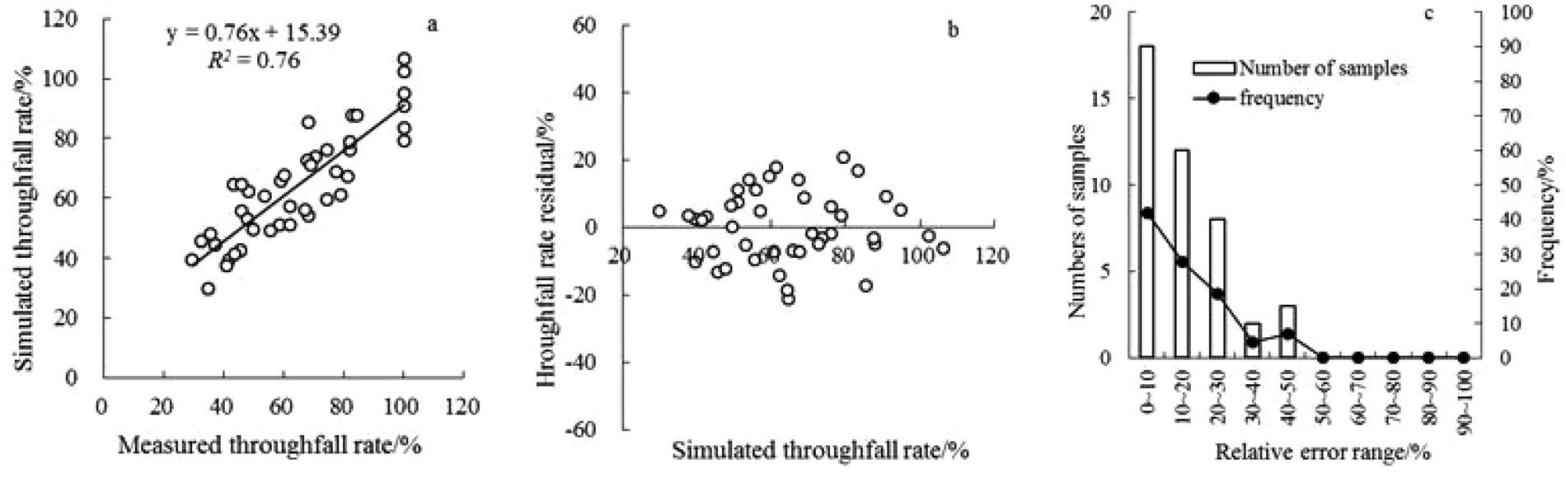
Fitting effect analysis of stepwise regression equation of the throughfall rate.

The linear stepwise regression equation resulted in a multiple correlation coefficient (R^2^) of 0.76 (Fig. 4a), but the relative error mean was 14.89%. The standard deviation was 11.65%, and the relative error of the larger observation sample size accounted for 30% (Fig. 4c). Clearly, it was not the best model.

**Figure 5:**
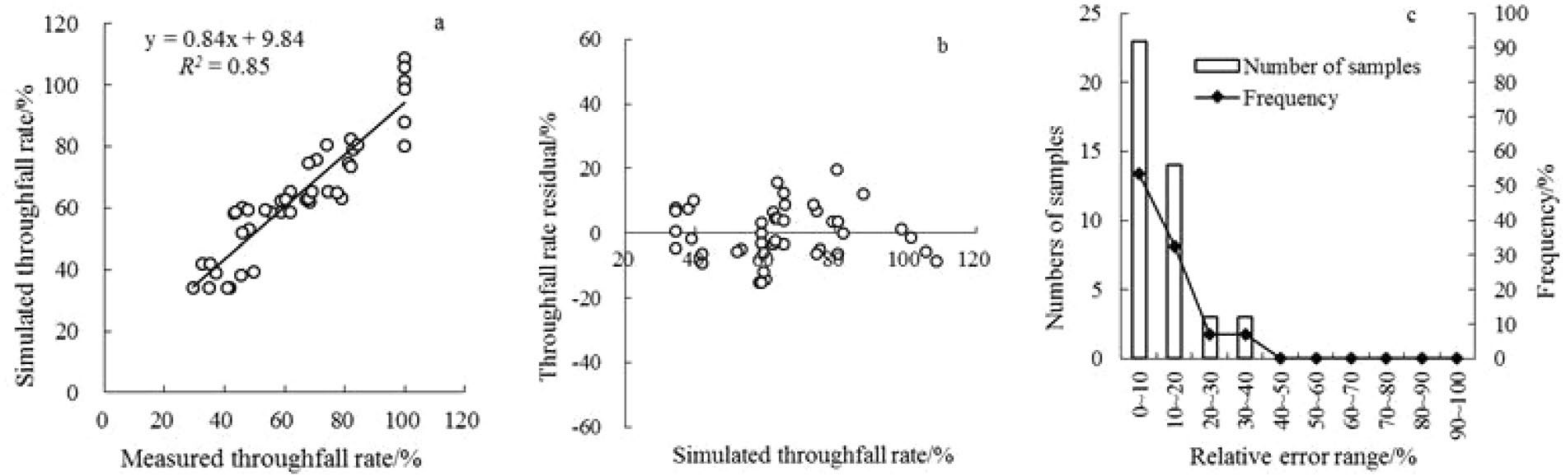
Analysis of the fitting effect of the quadratic response surface model for throughfall rate.

To generate a more accurate relationship, we fitted those factors that most significantly affected the throughfall rate – canopy density (X_6_) and rainfall class (X_7_) – using a quadratic response surface model (Table 5). The model multiple correlation coefficient increased to 85% (Fig. 5a), and the average relative error decreased to 11.74%. The standard deviation was 9.01% (Fig. 5c). Equation 3 is the throughfall rate for *P. tabulaeformis* according to the quadratic nonlinear model.

3 $${{\rm{Tf}}}_{r}( \% )=-436.68\cdot {{\rm{X}}}_{6}+51.43\cdot {{\rm{X}}}_{7}+192.18\cdot {{\rm{X}}}_{6}^{2}+8.73\cdot {{\rm{X}}}_{6}\cdot {{\rm{X}}}_{7}-11.87\cdot {{\rm{X}}}_{7}^{2}+230.58$$

**Table 5:** Results of the response surface quadratic (nonlinear) model for the throughfall rate in the *P. tabulaeformis* forest in the study area.

Regression	DF	Type I Sum of Squares	R-Square	F Value	Pr > F
Linear	2	13860.00	0.74	90.72	<0.0001
Quadratic	2	1927.10	0.10	12.61	<0.0001
Cross Product	1	37.87	0.0020	0.50	0.49
Total Model	5	15825.00	0.85	41.43	<0.0001
Residual	DF	Sum of Squares	Mean Square		
Total Error	37.00	2826.44	76.39		
Factor	DF	Sum of Squares	Mean Square	F Value	Pr > F
*X*_6_	3.00	10258.00	3419.47	44.76	<0.0001
*X*_7_	3.00	5147.46	1715.82	22.46	<0.0001
Parameter	DF	Estimate	Standard Error	T Value	Pr > |t|
Intercept	1	230.58	55.01	4.19	0.0002
*X*_6_	1	−436.87	127.01	−3.44	0.0015
*X*_7_	1	51.43	15.76	3.26	0.0024
*X*_6_ × *X*_6_	1	192.18	76.35	2.52	0.016
*X*_7_ × *X*_6_	1	8.73	12.41	0.70	0.49
*X*_7_ × *X*_7_	1	−11.87	2.81	−4.23	0.0001

The distribution of a random variable always involves a measure of randomness. When the residual plot is spread around e = 0, there is no bias. Cases where the standard deviation of the residual is not uniform represent a violation of homoscedasticity, and parametric regression cannot be used. This finding indicates that the model was not appropriate. The approximated residuals of the throughfall rate are scattered points and present a Λ-shaped distribution (Fig. 5b), indicating that the model was not suitable. By contrast, the residual plots of the quadratic response surface exhibit a random distribution. Thus, this model was relatively ideal (Fig. 5).

## Discussion and Conclusion

### Throughfall and rainfall

In this study, the throughfall rate of *P. tabulaeformis* increased with increasing rainfall, and the growth rate decreased gradually with increasing rainfall. When rainfall reached 25 mm, the throughfall rate was essentially stable, in agreement with the results of previous studies. CV_TF_ was strongly affected by rainfall at levels lower than 20 mm and was fairly stable when rainfall exceeded 20 mm (Fig. 4)^33–35^. The coefficient of variation of the rainfall rate and throughfall rate decreased with an increase in rainfall. The rate of decrease was initially fast and then became slow, and the turning point occurred at approximately 10 mm. This phenomenon was observed because at the beginning of a rainfall event, the majority of water saturates the canopy. The throughfall was initially low and then gradually increased before stabilizing after the canopy was completely saturated (Fig. 4).

The average CV_Tf_ values, i.e., 11–70%, were within the range of CV_Tf_ values (21–53%) measured in other forests and deciduous conifers. In this study, CV_Tf_ was higher than that reported for broadleaf forests in Japan, i.e., 12% for a mixed white oak forest^36^. When rainfall was heavier, the average CV_Tf_ values were higher; otherwise, the values were lower. These differences in the spatial variation of throughfall among stands can be expected due to differences in canopy species, canopy structure, density, spatial homogeneity and meteorological phenomena. Differences in experimental design, such as those related to the size of plots, number, size and spatial density of collectors, and sampling time scale, make it difficult to directly compare our results with those reported in previous studies. Nevertheless, it appears reasonable to expect a relationship between stand characteristics and the spatial variation of throughfall under the same climatic conditions.

### Factors that impact throughfall

The canopy features affecting throughfall include the following: the shape and size of trees, the roughness and thickness of the canopy, average tree height, average DBH, average branch height, stand density, branching pattern, leaf angle and LAI. Some studies have shown that the understory variation of throughfall and rainfall exhibit a negative correlation^37–40^. Studies have shown no significant correlation between throughfall and LAI^41,42^. In this study, the throughfall rate had a highly positive correlation with canopy density and rainfall, in agreement with previous studies^43,44^. The multiple correlation coefficient (R^2^ value) of the stepwise linear regression equation was 0.76. This finding indicates that canopy density, rainfall and height had important effects on throughfall. Interception losses almost always depend on rainfall type and other meteorological conditions^20^. Throughfall increases as canopy density decreases and increases with decreasing rainfall and height. Interception values increase with increasing canopy density and decrease with decreasing canopy density.

This study compared the relationship between throughfall and stand factors under different rainfall levels. A highly positive correlation between throughfall and rainfall level was found (Equations 2 and 3). At lower rainfall levels, rainfall interception increased rapidly with increasing rainfall. However, at higher rainfall levels, rainfall interception increased slowly with increasing rainfall, and the coefficient of variation of throughfall (CV_Tf_) decreased drastically, reflecting the limitations of canopy interception. A denser canopy resulted in larger interception and CV_Tf,_ with higher rainfall levels, but the impact weakened gradually.

When estimating the spatial variability of throughfall, the placement of water reservoirs was used as an index. The largest throughfall amounts were measured around the canopy edge, near the trunk, or midway between the trunk and canopy edge^45^. In this study, we chose a dense canopy, sparse canopy and forest gap to establish the water reservoirs. Doing so could decrease the variability of throughfall due to the position of reservoirs. The stepwise linear regression analysis equation indicated that there was a relationship between throughfall rate and tree height. Few studies exist on the relationship between throughfall rate and tree height. In this study, we found a limited relationship between the throughfall rate and tree height.
